# Variable body and tissue weight reporting in preclinical cachexia literature may alter study outcomes and interpretation

**DOI:** 10.1242/dmm.050148

**Published:** 2023-07-18

**Authors:** Anna G. Beaudry, Michelle L. Law

**Affiliations:** ^1^ Department of Health, Human Performance, and Recreation, Robbins College of Health and Human Sciences, Baylor University, Waco, TX 76706, USA; ^2^ Department of Human Sciences and Design, Robbins College of Health and Human Sciences, Baylor University, Waco, TX 76706, USA

**Keywords:** Preclinical, Data representation, Mouse model, Cancer cachexia, Colon-26 adenocarcinoma

## Abstract

Cancer cachexia is a multifactorial syndrome of body weight loss, muscle wasting and progressive functional decline, affecting many advanced cancer patients and leading to worsened clinical outcomes. Despite inherent limitations of many preclinical cachexia models, including large tumor burden, rapid tumor growth and young age of animals, these animal models are widely used and imperative for the study of cachexia mechanisms and experimental therapeutics. However, there are currently no guidelines for the reporting and representation of data in preclinical cachexia literature. We examined the current state of data reporting in publications using the colon-26 adenocarcinoma (C26) model of cachexia and compared statistical differences in reporting mechanisms using animals from our laboratory. We show that data reporting and representation in C26 preclinical cachexia literature are diverse, making comparison of study outcomes difficult. Further, different expression of body and tissue weights in our animals led to differential statistical significance, which could significantly alter data interpretation. This study highlights a need for consistent data reporting in preclinical cancer cachexia literature to effectively compare outcomes between studies and increase translatability to the human condition.

## Teaser

**Summary:** This study highlights the need for improved data reporting in preclinical cancer cachexia literature to effectively compare outcomes between studies and increase translatability to the human condition.

## INTRODUCTION

Cancer cachexia is a complex syndrome of bodily wasting and progressive functional decline (Baracos et al., 2018). Cachexia occurs in 30% of all cancer patients, and the risk for cachexia development is 70-90% for patients with cancers of the lung, liver and gastrointestinal tract (Anker et al., 2019). Cancer cachexia is thought to contribute to ∼20% of cancer deaths; however, this statistic may underestimate the true impact of cancer cachexia, as prevalence statistics are not currently included in the national cancer records of any country (Baracos et al., 2018; Tisdale, 2002). Patients with cancer cachexia often experience profound fatigue and weakness (Zhou et al., 2017), reduced anticancer therapy tolerance and effectiveness (Aapro et al., 2014; Muscaritoli et al., 2016; Ross et al., 2004; Vaughan and Martin, 2022), decreased quality of life (Baracos et al., 2018; Fearon et al., 2006; Wallengren et al., 2013) and increased mortality (Baracos et al., 2018; Fearon et al., 2011, 2006; Penet and Bhujwalla, 2015; Wallengren et al., 2013). Heightened risk of cancer-related medical complications and increased medical costs have also been documented in this patient population (Arthur et al., 2016; Gourin et al., 2014). Limited therapeutic options exist for cachexia patients.

The use of preclinical animal models has greatly enhanced our understanding of cancer cachexia pathophysiology and allows for the avoidance of limitations faced in clinical research. Preclinical cancer cachexia models are valuable for the exploration of mechanistic questions and the assessment of experimental therapeutics. Multiple categories of preclinical animal models have been developed for the study of cancer cachexia, including ectopic or orthotopic tumor cell implantation, human cancer cell or patient-derived xenografting, and spontaneous tumor growth models in genetically engineered mice (Ballarò et al., 2016).

Ectopic tumor cell implantation models are currently the most commonly used in cachexia literature. These models involve subcutaneous, intramuscular or intraperitoneal injection of syngeneic rodent cancer cells into the experimental animal, followed by rapid tumor growth and the inflammatory cascade that accompanies it. Commonly used models in this category are colon-26 adenocarcinoma (C26), Lewis lung carcinoma (LLC), MAC 16 adenocarcinoma, B16 melanoma, Walker 256 carcinosarcoma, and Yoshida ascites hepatoma 130 (AH130) (Ballarò et al., 2016; Penna et al., 2016). Rudimentary characteristics of this category of preclinical cancer cachexia models that limit translatability include lack of natural tumor development, heterogeneity of tumor growth amongst experimental animals, ectopic tumor placement, large tumor burden, accelerated cachexia development and progression, decreased age of host, lack of metastasis, and absence of anti-cancer therapies, compared with most humans with cancer. Despite limitations, ectopic syngeneic tumor cell implantation models remain widely used due to their relative ease of use, rapid tumor development, and subsequent manifestation of body weight loss and muscle wasting. Additionally, they are well characterized in the published literature. Nonetheless, these models require careful study conduction and data representation to enhance replication and translatability.

After its genesis in 1975, the C26 model of cancer cachexia became prominent in the literature in the 1990s (Aulino et al., 2010; Corbett et al., 1975). Throughout the past three decades, this classical model of cancer cachexia has been used in over 246 experimental publications, with exponential growth in publication numbers over the past decade. The C26 model utilizes BALB/c or CD2F1 (F1 hybrid of female BALB/c and male DBA/2) mice. Study initiation often occurs at ∼6-8 weeks of age, when C26 cells are subcutaneously injected. Tumors are visible after 7-14 days, and significant body weight loss is achieved after ∼16-27 days. This cancer model induces cachexia through tumor growth and systemic inflammation, leading to muscle and adipose wasting. Anorexia likely contributes to negative energy balance and body weight loss towards the end of the study (Asp et al., 2011; Flint et al., 2016), but decreased food intake alone is insufficient to induce lean tissue wasting, as described in pair-feeding experiments (Tian et al., 2010). Body and tissue wasting occurs predominantly from increased lipid and protein catabolism, the latter attributed to increased activity of the ubiquitin–proteasome system (Aulino et al., 2010) and autophagy (Penna et al., 2013, 2019), as well as decreased protein synthesis (Argilés et al., 2014; Smith and Tisdale, 1993). Splenomegaly and elevated circulating levels of pro-inflammatory cytokines, namely high levels of IL-6, are observed (Hetzler et al., 2015; Narsale and Carson, 2014; Strassmann et al., 1992).

Clinically, the diagnostic criteria for cachexia are (1) body weight loss >5% within 6 months, or (2) >2% weight loss in individuals with a body mass index <20 kg/m^2^ or with an appendicular skeletal muscle index consistent with sarcopenia (Fearon et al., 2011). Although there are certainly other symptoms and biomarkers that can predict cachexia severity in patients (e.g. fatigue, decreased quality of life and mobility/activity, insulin resistance, and circulating inflammatory mediators and tumor-derived factors), body weight and lean tissue loss remain key defining features of patients with cancer cachexia. As such, accurate and thorough reporting of animal body and tissue weight in the preclinical literature is a necessary component for assessing cachexia severity, comparing results across studies and improving translatability to the human condition. Nonetheless, at present, there are no guidelines for methodological and data reporting procedures in preclinical cancer cachexia research. Numerous methods of body and tissue weight reporting are currently used, and these methods may provide inconsistent information to readers. Given the wide utilization and necessity of preclinical cancer cachexia models, coupled with the inherent limitations of these models, particularly large tumor burden, rapid tumor growth, and use of young animals that may still be growing, the development of guidelines for methodological and data reporting procedures would benefit the cachexia research community by improving the transparency and interpretation of research results. As expressed by the Animal Research: Reporting of *In Vivo* Experiments (ARRIVE) Guidelines 2.0 (du Sert et al., 2020a,b), appropriate reporting and representation of methodological procedures and transparent data reporting are essential for study interpretation and translatability.

**Fig. 1.**
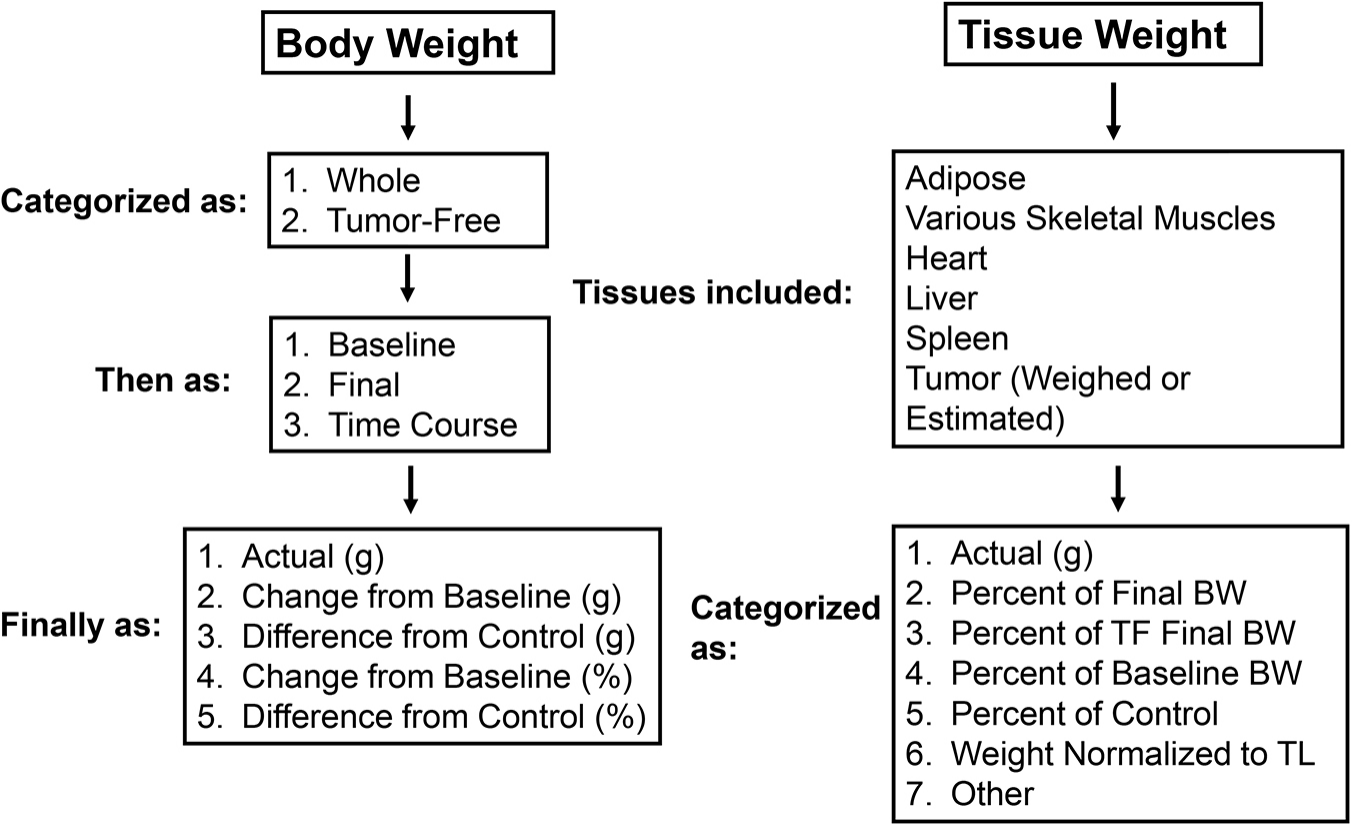
**Body and tissue weight reporting categories captured during literature data extraction.** BW, body weight; TF, tumor-free; TL, tibia length.

Before recommendations for reporting body and tissue weight can be developed, current practices in data reporting must be assessed. Therefore, the purpose of the present study was to (1) identify and quantify different mechanisms (Fig. 1) for reporting body and tissue weights in the published literature using the C26 model, and (2) apply these reporting mechanisms to data from our laboratory to identify whether differences exist in statistical significance between data presentations. We show here a wide variation in the types of data reported and the presentation of data in C26 studies. When applied to our animals, different data reporting mechanisms yield differences in statistical significance between mice with and without tumors. These results have important implications for the interpretation of preclinical cancer cachexia research and highlight a need for the cancer cachexia research community to develop guidelines for data reporting to enhance the replicability and translatability of research results.

## RESULTS

### Publication characteristics

**Fig. 2.**
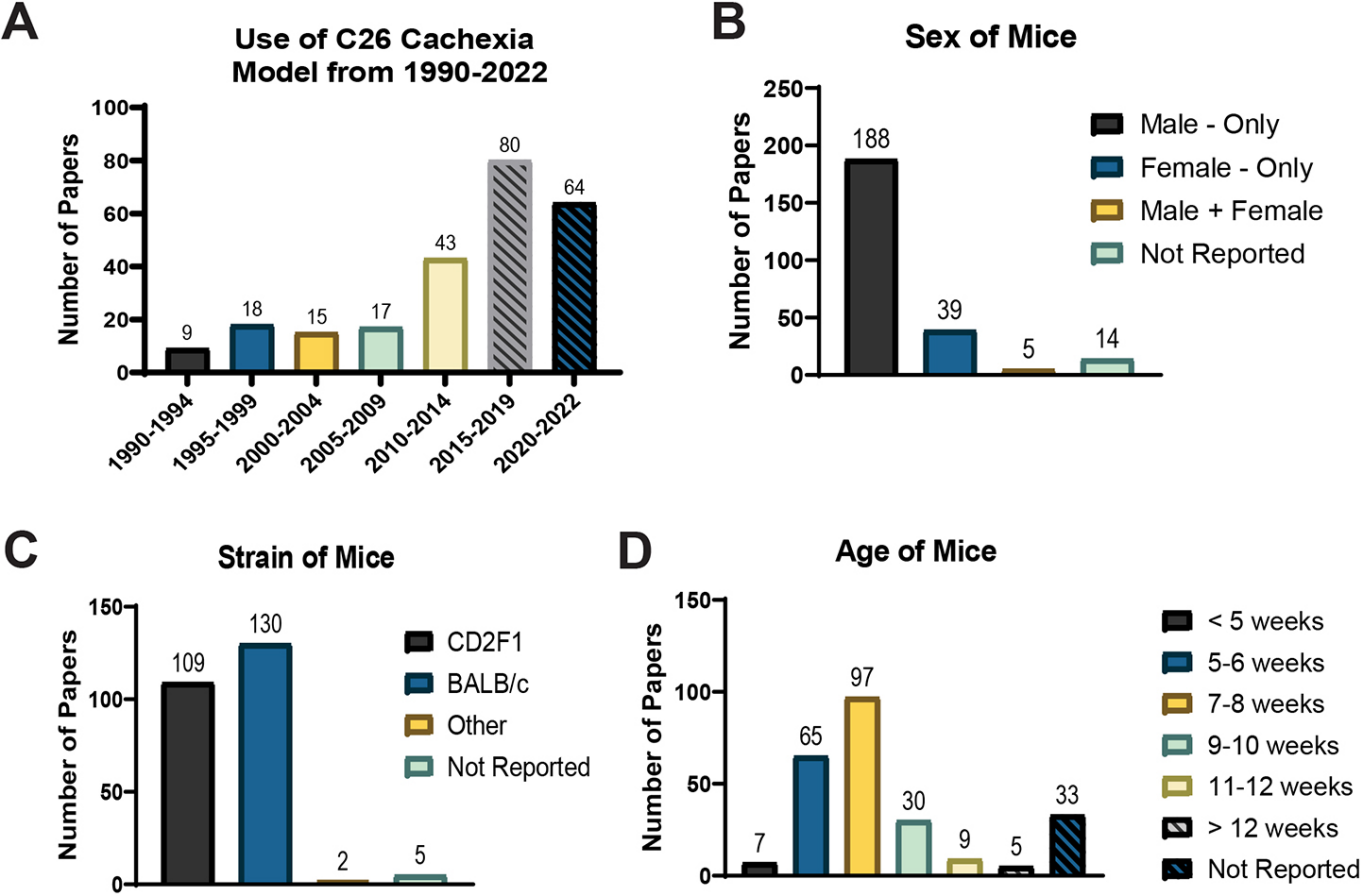
**Publication characteristics.** (A) Use of the colon-26 adenocarcinoma (C26) preclinical cancer cachexia model over the past three decades, from 1990 to 2022. (B-D) Sex (B), strain (C) and age (D) of mice used in C26 publications.

A total of 246 publications were assessed. Of these, 59% were published within the past 7 years (2015-2022) (Fig. 2A). Most publications (76%, *n*=188) used male mice only (Fig. 2B). Regarding the strains of mice used for experiments, 109 used CD2F1 and 130 used BALB/c (Fig. 2C). Ages of mice varied, with 5-6 weeks and 7-8 weeks of age being the most frequent age ranges (Fig. 2D).

### Weight reporting mechanisms in published literature

#### Body weight

**Fig. 3.**
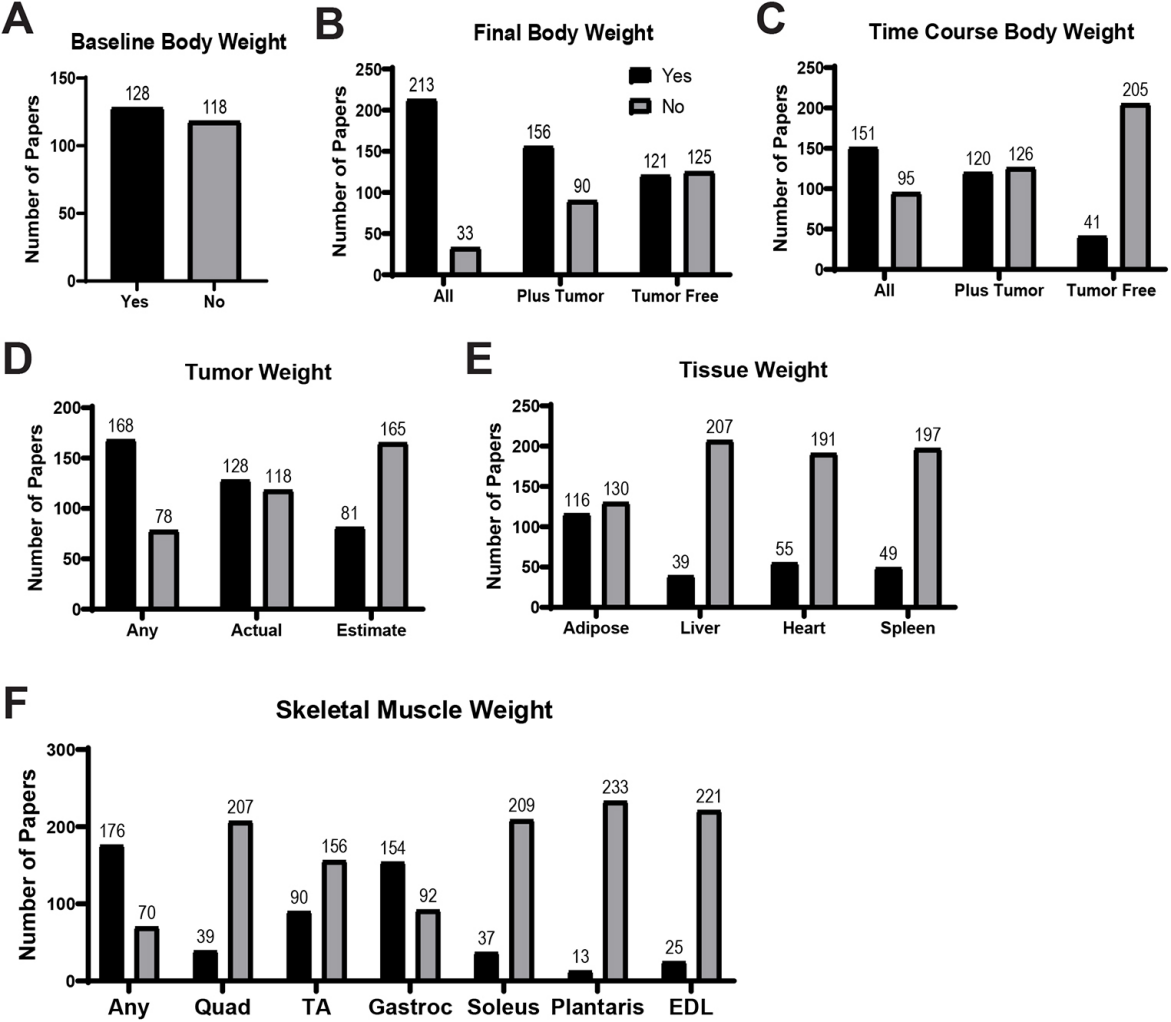
**Body and tissue weight reporting frequencies from all 246 publications.** ‘Yes’ represents the number of publications that reported the given variable using at least one reporting mechanism (actual, change from baseline, percentage of final body weight, etc.). (A) Body weight of animals reported in any manner when the experiment began. (B) Final body weight reported in any manner, as whole-body weight (including tumor) on necropsy day, or as tumor-free body weight on necropsy day. (C) Time course body weight reported in any manner, as whole-body weight (including tumor) of animals throughout the course of experimental days, or as tumor-free body weight (using tumor volume estimation) of animals throughout the course of experimental days. (D) Tumor weight reported in any manner, actual (extracted and weighed at time of sacrifice) or estimated based on measured tumor volume. (E) Tissue weights. (F) Skeletal muscle weights. Any, papers that included any muscle weight; EDL, extensor digitorum longus; Gastroc, gastrocnemius; Quad, quadricep; TA, tibialis anterior.

Baseline body weight was reported in 52.0% of publications (Fig. 3A). Final body weight was reported by some method in 86.5% of publications. Body weight including tumor weight was reported in 63.4% of publications, and tumor-free final body weight was reported in 49.2% of publications (Fig. 3B). Time course weight, body weight reported throughout the study period, was reported by some method in 61.4% of publications. Time course weight including tumor weight was reported by 48.8% of publications, and time course weight with tumor weight removed via tumor volume calculation was reported in 16.7% of publications (Fig. 3C).

#### Tissue weights

Tumor weight was reported by some method in 68.3% of publications, as actual weight by 52% of publications and as estimated weight by 32.9% of publications (Fig. 3D). At least one form of skeletal muscle weight reporting was included in 71.5% of publications. The percentage of publications that reported the following tissue and skeletal muscle weights are as follows: adipose tissue, 47.2%; liver, 15.9%; heart, 22.4%; spleen, 19.9%; quadriceps, 15.9%; tibialis anterior, 36.6%; gastrocnemius, 62.6%; soleus, 15%; plantaris, 5.3%; and extensor digitorum longus, 10.2% (Fig. 3E,F). Of all 246 studies, 25 (10%) did not report any type of body weight or muscle weight data.

Beyond weight reporting category (i.e. baseline body weight, final body weight, muscle and organ weights, etc.), the manner in which data were reported within each category varied. Within body weight reporting categories, body weight was reported as grams, change from baseline weight (grams or as a percentage), or as the difference from tumor-free control (grams or as a percentage) (Table 1). Tissue weight categories were reported as weight in grams, percentage of final body weight (including or not including tumor mass), percentage of baseline weight, percentage of tumor-free control animal tissue weight, or weight normalized to tibia length (Table 2). For body and tissue weight, actual weight was the most commonly reported. Secondarily, body weight expressed as a percentage change from baseline weight and tissue weights as a percentage of the control animal tissue weights were most prevalent.

**Table 1.**
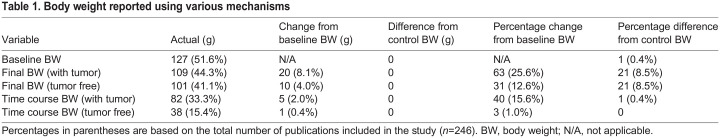
Body weight reported using various mechanisms

**Table 2.**
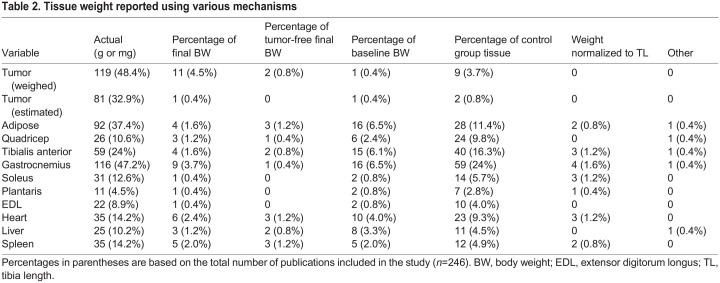
Tissue weight reported using various mechanisms

### Examination of weight reporting mechanisms from published literature using animals from our laboratory

#### Body weight

**Fig. 4.**
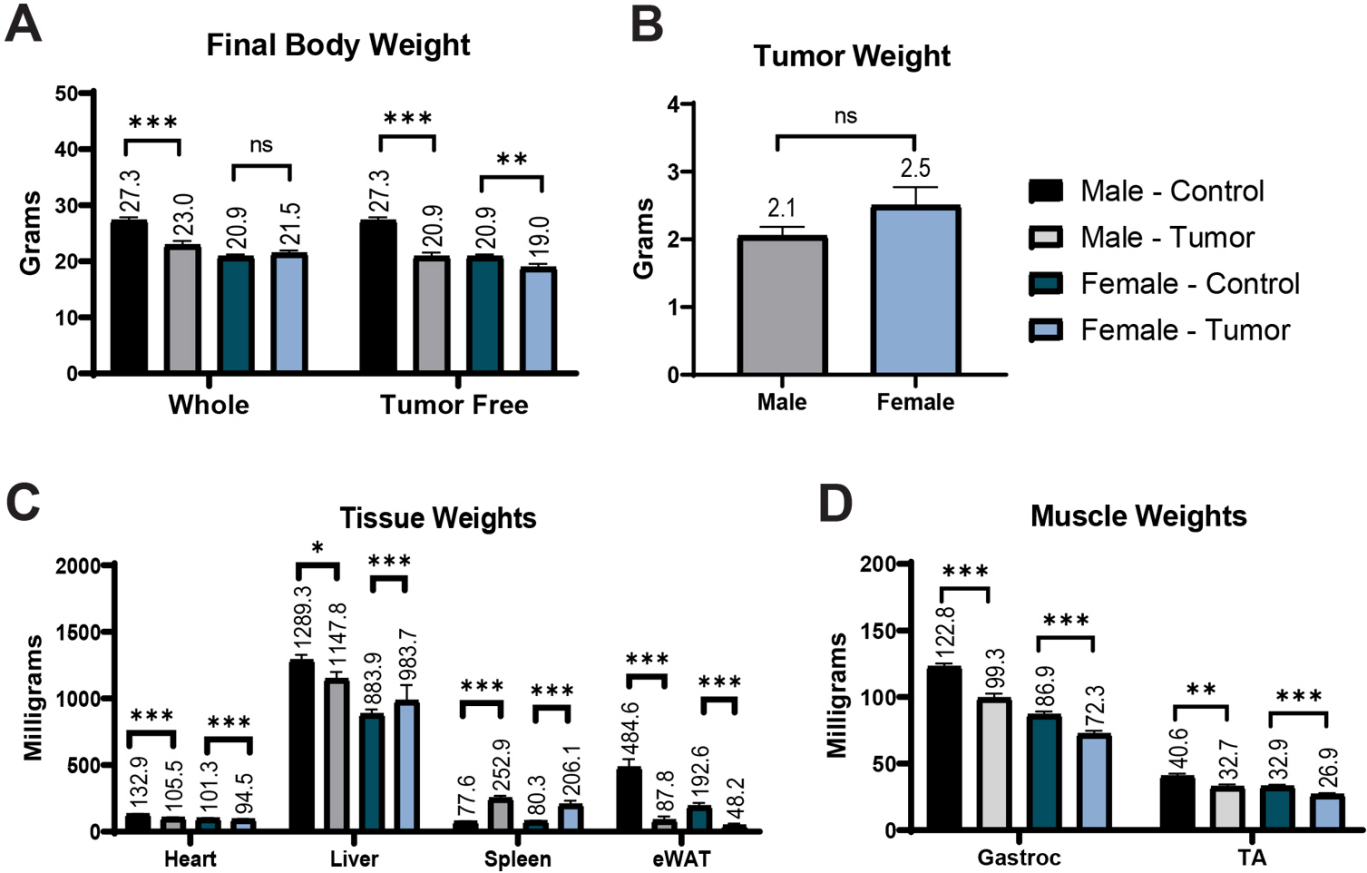
**Body and tissue weight data from our laboratory.** (A) Final body weight expressed as whole-body weight (plus tumor) and tumor-free body weight of male and female animals. (B) Tumor weight. (C) Tissue weights. (D) Muscle weights. eWAT, epidydimal white adipose tissue; Gastroc, gastrocnemius; TA, tibialis anterior. Male control, *n*=13; female control, *n*=13; male tumor, *n*=14; female tumor, *n*=12. Data are mean±s.e.m.; ns, not significant; *P<0.05, **P<0.01, ***P<0.001 compared to control (unpaired, two-tailed *t*-test).

Upon sacrifice, the whole-body weight of male tumor-bearing mice (23.0 g) was 16.2% less than that of male control mice (27.3 g), and the tumor-free final body weight of male tumor-bearing mice (20.9 g) was 23.5% less than that of the male control mice. The whole-body weight of female tumor-bearing mice (21.5 g) was 103.2% of that of female control mice (20.9 g), but when tumor mass was subtracted, female tumor-bearing mice (18.9 g) weighed 9% less than female control mice (Fig. 4A). All male tumor-bearing final body weight values with and without tumor (actual, change from baseline, change from maximum weight, difference from control) were significantly different from male control animal values (*P*<0.001) (Table 3). When comparing the final body weight values of female tumor-bearing animals with those of female control animals (actual, change from baseline, change from maximum weight, difference from control), the only statistically significant value was the change from maximum body weight (control, −0.4 g; tumor, −1.4 g; *P*=0.004). However, tumor-free final body weight values, represented as actual, change from baseline, change from maximum weight and difference from control, were all significantly different from female control animal values (*P*=0.01 or *P*<0.001) (Table 3).

**Table 3.**
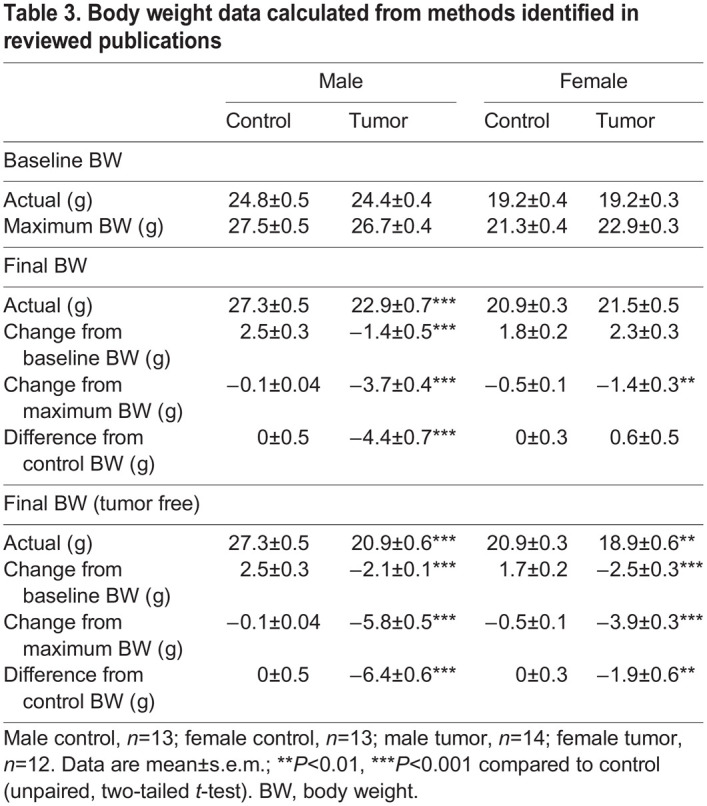
Body weight data calculated from methods identified in reviewed publications

#### Tissue weights

Actual tumor weight was non-significantly different between female tumor-bearing mice and male tumor-bearing mice (Fig. 4B). Tumor weight expressed as a percentage of tumor-free final body weight was 13.6% for female mice and 9.9% for male mice, and the difference between these percentages was statistically significant (*P*<0.05) (Table 4). Heart weight of male tumor-bearing mice (105.5 mg) was significantly lower than that of male control mice (132.9 mg) (*P*<0.001) (Fig. 4C). Heart weight expressed as a percentage of final body weight in male tumor-bearing mice (0.46%) was significantly different from that in male control mice (0.49%) (*P=*0.016), but when tumor weight was subtracted, the difference between the percentages was not significant (control, 0.49%; tumor, 0.51%; *P*=0.070) (Table 4). Heart weight of female tumor-bearing mice (94.5 mg) was significantly lower than that of female control mice (101.3 mg) (*P*<0.001) (Fig. 4C). Heart weight expressed as a percentage of final body weight in female tumor-bearing mice (0.44%) was significantly different from that in female control mice (0.49%) (*P<*0.001), but when tumor weight was subtracted, the difference between the percentages was not significant (control, 0.49%; tumor, 0.50%; *P*=0.581) (Table 4). Gastrocnemius weight of male tumor-bearing mice (99.3 mg) was significantly lower than that of male control mice (122.8 mg) (*P*<0.001) (Fig. 4D). Gastrocnemius weight expressed as a percentage of final body weight in male tumor-bearing mice (0.43%) was not significantly different from that in male control mice (0.45%) (*P=*0.115), but when tumor weight was subtracted, a significant difference existed, with tumor-bearing animals having a larger percentage than that of controls (control, 0.45%; tumor, 0.48%; *P*=0.018) (Table 4). Gastrocnemius weight of female tumor-bearing mice (72.3 mg) was significantly lower than that of female control mice (86.9 mg) (*P*<0.001) (Fig. 4D). Gastrocnemius weight expressed as a percentage of final body weight in female tumor-bearing mice (0.34%) was also significantly lower than that in female control mice (0.42%) (*P<*0.001). Likewise, when tumor weight was subtracted, a significant difference remained, with tumor-bearing animals having a smaller percentage than that of controls (control, 0.42%; tumor, 0.38%; *P*=0.018) (Table 4). Additional statistical differences in tibialis anterior and liver weights can be found in Table 4 and Fig. 4. Owing to massively increased spleen weight and decreased adipose weight, the reporting methodology did not yield differences in statistical significance for these tissues (Table 4 and Fig. 4).

**Table 4.**
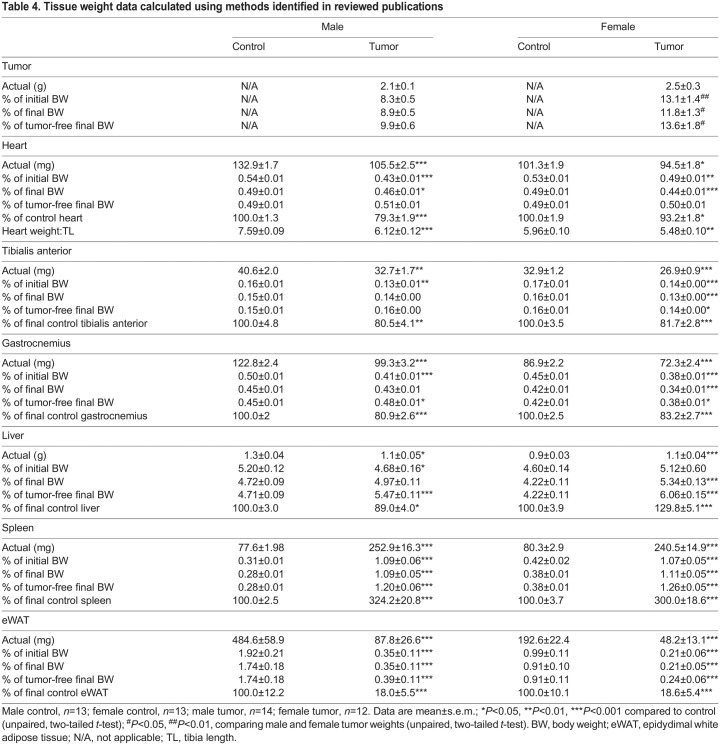
Tissue weight data calculated using methods identified in reviewed publications

## DISCUSSION

In this study, we extracted data from all publications in the PubMed database using the C26 cachexia model from 1990 to 2022 to quantify the prevalence and types of reporting for body and tissue weights. We followed this analysis with data from our own laboratory to examine differences in statistical outcomes using the reporting methodologies identified in our data extraction. We found variability in the body and tissue weight data reported, and in the presentation of these data. We found differential effects on statistical significance using various reporting methodologies with our own data, particularly related to whole-body weight and tumor-free body weight and use in the presentation of tissue weights. This is the first, to our knowledge, comprehensive analysis of body and tissue weight reporting mechanisms in the cachexia literature. Our findings have important implications for the interpretation and replicability of preclinical cancer cachexia studies.

At present, the cancer cachexia literature is inconsistent in terms of the data reported and how these data are reported in preclinical studies. The present study reveals that 48.0% and 13.4% of studies did not report any type of baseline and final body weight of animals, respectively. These data points are imperative for confirming the existence and severity of cachexia in experimental animals. Fifty-two percent of publications reported actual tumor weight upon study conclusion. This is also an important finding to note, as lack of tumor weight reporting does not allow for the comparison of tumor burden across studies. Further, less than half of publications reported any form of adipose weight, only 71.5% of publications reported any form of skeletal muscle weight, and 10% of publications did not report any type of body or muscle weight data. Body weight loss and wasting of skeletal muscle and adipose tissue are cornerstones of cachexia pathology, and, thus, thorough reporting of these measurements is needed for accurate assessment of cachexia severity.

Beyond reporting body and tissue weights, much variability was observed regarding the manner in which this information was represented. Body weight was reported as grams, change from baseline (grams or as a percentage), or as the difference from tumor-free control (grams or as a percentage). Tissue weights were reported as weight in grams or milligrams, percentage of final body weight (including or not including tumor), percentage of baseline body weight, percentage of tumor-free control tissue weight, or weight normalized to tibia length. Tumor weight was reported as actual or estimated tumor volume. Within these two categories, tumor weight was reported as weight in grams, percentage of final body weight (including or not including tumor), or percentage of baseline body weight. The present study using data from our laboratory demonstrates that these various mechanisms of data reporting can result in varied statistical significance within the same cohort of animals. For example, comparing our male tumor-bearing mice to male control mice resulted in statistical significance in all categories of final body weight including tumor (actual, change from baseline, change from maximum weight, difference from control), whereas data from female tumor-bearing mice compared with those from female control mice only demonstrated statistical significance for final body weight including tumor when it was expressed as change from maximum weight. However, when the tumor weight was removed from the final weight, the comparison of both male and female tumor-bearing animals to controls displayed statistical significance in all tumor-free final body weight measures. Similar trends were discovered when exploring tissue weight measures. For example, actual gastrocnemius weight was statistically different when comparing tumor and control animals of both sexes, but was no longer significant in male animals when expressed as a percentage of final body weight. Thus, although the inclusion of these various reporting mechanisms and data presentations can be helpful in the context of individual studies, the selection of one over the other without foundational reporting (such as actual weight in grams or milligrams) may introduce selection bias, thus misleading readers and future compounding work.

First developed nearly 50 years ago, the C26 model remains one of the most common and well-characterized models to study cachexia. Despite this, notable variability exists between studies related to study timeline, tumor size, and severity of body weight loss and tissue atrophy. Identifying contributing factors to this variability is beyond the scope of this study, but may include the source of C26 cells, cell culturing techniques, age and strain of animals, sex, tumor size, and additional procedures and handling of animals done over the duration of the study. It is challenging to evaluate and compare studies with different experimental timelines and variable tumor sizes, or those that use mice at a variety of growth stages. To give context to this point, suppose that one study had an 18-day experimental timeline with 3 g tumors and a −4.0 g change from baseline in tumor-free body weight, while another study had a 26-day experimental timeline with 1.5 g tumors and a −1.0 g change from baseline in tumor-free body weight. Without the inclusion of these foundational weight data, the reader may incorrectly assume that the study with more experimental days would result in a greater tumor burden and increased weight loss. If actual body and tumor weights are not expressed in grams, the severity of cachexia in these two studies may be difficult to assess, especially in the context of variable ages and baseline weights.

It is also important to consider data representation in studies with less severe cachexia phenotypes, or early-stage studies, in which weight changes are more nuanced. The female tumor-bearing animals from our laboratory used in this study, although not sacrificed at an early-stage cachexia timepoint, demonstrated milder wasting than did their male tumor-bearing counterparts. Although, on necropsy day, female tumor-bearing animals (21.5 g) weighed more than female control animals (20.9 g), their tumor-free final body weight (18.9 g) was 2 g less than that of female controls (20.9 g). Thus, there are large differences in data presentation and statistical significance, in this case when representing final body weight as whole (including tumor) or as tumor free. Likewise, this then also impacts the presentation and statistical significance of tissue weights if they are reported as a percentage of final body weight (whole or tumor free). It is logical to assume that this is not only true of male versus female mouse cancer cachexia data but would also be observed in early-stage studies in which the cachexia phenotype is less severe.

The present study also revealed, somewhat unsurprisingly, that a sex bias still exists in preclinical cancer cachexia research. Most studies included male animals alone. Fourteen studies failed to report the sex of animals used. Of these 14 publications, one was from 1990-2000, two were from 2001-2010, and 11 were from 2011-2022. To our knowledge, only five studies performed within the dates of our literature search using the C26 model of cancer cachexia used both male and female animals. Of these studies, only two performed an actual head-to-head comparison (other works combined male and female animals for analysis). Sex differences in cancer cachexia susceptibility, mechanistic proregression and outcomes are known to occur (Hetzler et al., 2015; Montalvo et al., 2018; Rosa-Caldwell et al., 2021; Zhong et al., 2022; Zhong and Zimmers, 2020). Thus, appropriate inclusion, analysis and transparent reporting of sex in preclinical studies is imperative. Strain of mice and animal age upon study initiation were also found to vary amongst publications, potentially further limiting study comparison and translatability. Apart from a few publications, the strain of animals used was divided between CD2F1 (44.3%) and BALB/c (52.8%). Of all publications, 13.4% did not include the age of animals used. Of these 33 studies, three were from 1990-2000, six were from 2001-2010, and 24 were from 2011-2022. Age is an important piece of information, particularly as animals younger than 8 weeks are still growing (Fox, 2007; Jackson et al., 2017). In our study, including animals between 8 and 10 weeks of age at baseline, weight gain was seen throughout the course of the study, as evidenced by differences in baseline and maximum weight. Growth and weight gain are important considerations when studying mechanisms of weight loss and muscle wasting. Significant weight gain of control animals over the course of the study can further complicate interpretation of cachexia severity. For example, expressing weight loss in tumor-bearing animals as a percentage of that of control animals at study termination, baseline body weight or maximum body weight may yield differential results and complicate data interpretation.

Not all preclinical cancer cachexia publications are the same (i.e. not every experiment is testing the ability of drug ‘x’ to decrease skeletal muscle wasting), but consistent body and tissue weight data presentation across preclinical works is necessary. This is especially important because hallmark features of cachexia in preclinical and clinical settings include loss of body weight and lean tissue. Accurate and thorough reporting of animal weight in the preclinical literature is crucial for interpretation of study results within the preclinical research community, and for translatability to the human condition, which must always be the bedrock of preclinical work. It seems appropriate, then, to suggest that all preclinical cancer cachexia publications (beyond just those that use the C26 cell line) clearly represent data that confirm whether and to what degree the animals are cachectic. At a minimum, the reporting of a few fundamental data points would provide consistent information to give readership insight into the cachectic state of the animals in that study. We suggest that these include the following measurements of both the control and experimental groups: baseline body weight in grams, final body weight in grams as both whole and tumor-free weight, actual tumor weight in grams weighed at time of necropsy, spleen weight in grams/milligrams, the weight of one adipose depot in grams/milligrams, and the weight of at least one skeletal muscle in grams/milligrams. Although normalized body and tissue weights can provide additional insight to readers, these representations should not replace the reporting of actual weights, particularly due to challenges with statistical analysis of ratios (Allison et al., 1995). We also suggest that, at a minimum, animal sex, strain and age at time of inoculation, as well as the number of study days completed prior to necropsy, are included. Studies utilizing cell lines that generate increased levels of IL-6 or other inflammatory markers should also consider including these measures. This certainly is not an exhaustive list, but one that should serve as a reporting baseline. This information is important not only for the benefit of the reader but also for reporting consistency across the preclinical cancer cachexia research community. Betancourt and colleagues recently developed an animal cachexia score (ACASCO) that can be used to determine an animal's stage along the cachexia continuum and as a primary endpoint in preclinical cancer cachexia therapeutic research (Betancourt et al., 2019). Although it is not practical for all preclinical cancer cachexia studies, the ACASCO should be considered for use in those studies testing experimental therapeutics.

This study was not without limitation. First, in terms of data extraction from publications, we included a reporting mechanism as ‘yes’ if it was reported once. However, there were occasions when multiple experiments were included in one publication, and sometimes different results and reporting mechanisms were included in the different experiments. This was not captured in our analysis, and thus our study may have overrepresented data reported in individual experiments within publications. Second, our data extraction was limited to the C26 model of cachexia. However, the C26 model has many similarities to other ectopic tumor models, thus making our recommendations relevant to a large proportion of the preclinical cachexia literature. Third, we did not include analysis of how well other factors such as age, sex, strain, cell line clone, single-nucleotide polymorphisms in cell line, source of animals and cells, protocol for cell preparation, number of cells injected, mouse housing characteristics, inflammation, or other biomarkers impact disease progression, predict for cachexia severity, and/or increase variability between studies. Fourth, we do not propose ‘diagnostic’ criteria or defining features of cachexia that should be evident in all preclinical models. Identifying predictive biomarkers for improving the clinical diagnosis of cachexia and developing models with improved clinical translatability are major topics of discussion and research in the cachexia field and are beyond the scope of the present work. The goal of this study was to assess variability in reporting of an important feature of cachexia (body and tissue wasting) and how this variability can affect data interpretation in the C26 model. We acknowledge that biochemical markers, muscle strength and physical functioning, among other factors, are indeed important components of assessing cachexia development and progression and should be included in publications whenever possible. Finally, the second PubMed data search took place in May 2022, so any publications that were produced after this time were not included in our analysis.

The present study shows that body and tissue weight reporting and data representation in C26 preclinical cancer cachexia research are currently inconsistent, making the interpretation and comparison of study outcomes difficult. Despite inherent limitations with C26 and other ectopic tumor-induced cachexia models, these models remain the most predominant and thoroughly characterized for the study of cachexia, further necessitating the standardization of data representation. The inclusion of key methodological details, as well as the transparent reporting of results, influences the work's meaningfulness and clinical translatability. With the omission of foundational details, readers may incorrectly interpret findings and take next steps that are not prudent. Even worse, discoveries may not be applicable to the human condition. This study highlights a need to develop guidelines for data reporting in preclinical cachexia literature to effectively compare outcomes between studies and increase clinical translatability.

## MATERIALS AND METHODS

### Literature data extraction

In May 2021, a PubMed literature search was conducted. The following search terms were used: ‘cachexia [Mesh] AND (colon-26 OR C26 OR colon 26 OR CT26)’ and ‘(cachexia AND (colon-26 OR C26 OR colon 26 OR CT26)) NOT MEDLINE’. A secondary search using the same search terms was completed in May 2022 to identify new papers published during the past year. From the combination of these searches, 285 publications were identified and analyzed. Of these, 39 publications were eliminated due to lack of fit. Publications not written in English (*n*=4), literature reviews (*n*=3), those only using C26 cells in culture (*n*=10), those that did not include cancer cachexia or mice (*n*=8), duplicates (*n*=13) and retractions (*n*=1) were excluded from the analysis. Publications that did fit the search criteria, a total of 246, were assessed.

Each publication was evaluated independently by two reviewers (A.G.B. and M.L.L.), and discrepancies between reviewers' data were discussed to reach consensus. Weight reporting mechanisms used to report body and tissue weights were recorded. Body weight was categorized as either baseline weight, tumor-free or tumor-bearing final body weight, or tumor-free or tumor-bearing time course weight. Publications were counted as recording time course weight if they included any additional body weight measurements beyond baseline and final. Within these categories, body weight was reported as actual weight in grams, change from baseline weight (grams or as a percentage), or as the difference from tumor-free control animals (grams or as a percentage). Tissues included adipose, various skeletal muscles, heart, liver and spleen. Tissue weight reporting mechanisms captured included weight in grams or milligrams, percentage of final body weight (including or not including tumor), percentage of baseline body weight, percentage of control group tissue weight, or weight normalized to tibia length. Tumor weight reported as actual or estimated was also captured. Actual tumor weight refers to the weight of the tumor as measured via dissection on necropsy day; estimated tumor weight refers to estimated tumor volume measured by calipers. Sex, strain and age of mice, as well as the date of publication, were also captured. A schematic to illustrate the data collected is shown in Fig. 1. Dataset 1 provides the raw data extracted from each paper.

### Live-animal portion of study

To compare data reporting methods for body and tissue weights found in our literature search, a study was conducted using the C26 cachexia model in our laboratory. All methods were approved by the Institutional Animal Care and Use Committee at Baylor University.

#### Cell culture

C26 cells were obtained from the Division of Cancer Treatment and Diagnosis Tumor Repository, National Cancer Institute (Frederick, MD, USA), where they had been tested for purity and contamination. Cells were cultured in RPMI 1640 medium (Gibco, Waltham, MA, USA) supplemented with 5% fetal bovine serum, 100 U/ml penicillin and 100 μg/ml streptomycin at 37°C in a 5% CO_2_ humidified atmosphere. Subconfluent (∼75%), low-passage (≤6 passages from attainment) cells were trypsinized, centrifuged, counted and resuspended in sterile phosphate-buffered saline (PBS) at a concentration of 1×10^7^ cells/ml immediately before implantation.

#### Animals

Eight- to 10-week-old male (*n*=27) and female (*n*=27) CD2F1 (F1 hybrid of female BALB/c and male DBA/2) mice were included in this study. Sample size was determined based on previous literature (Asp et al., 2011). The mice were kept on a 12:12 h light–dark cycle with *ad libitum* access to standard rodent chow and water. Mice were randomly assigned (by cage) to tumor or control groups. Male (*n*=14) and female (*n*=14) mice were inoculated with 1×10^6^ C26 cells in 100 µl PBS, and the remaining mice received an equal volume of sterile PBS via subcutaneous injection in the left flank. Injections were performed under isoflurane anesthesia. Body weight, body condition, food intake and tumor dimensions were assessed regularly. Mice that met humane endpoint criteria prior to the end of the study (*n*=2 females with tumors) were excluded from analysis. Mice were sacrificed 25-27 days after tumor cell injection. Each mouse was placed under isoflurane general anesthesia (5% in oxygen), and, after non-response to a firm toe pinch, blood was drawn through cardiac puncture. Mice were then euthanized via terminal thoracotomy. Tissue excision and organ collection were performed. Collected tissues were weighed and snap-frozen in liquid nitrogen, then stored at −80°C. Body and tissue weight raw data from this study are found in Dataset 2.

### Statistical analysis

Unpaired, two-tailed *t*-tests were used to compare male tumor-bearing animals with male control animals, and female tumor-bearing animals with female control animals. An unpaired, two-tailed *t*-test was also used to compare male tumor weight with female tumor weight. Assumptions of independence, normality, homogeneity of variance and random sampling were all met for each *t*-test. Significance was set at *P*<0.05. All data were analyzed using the IBM Statistical Package for the Social Sciences 28 (IBM SPSS Statistics, Cary, NC, USA), and figures were compiled using GraphPad Prism 9 (La Jolla, CA, USA). Data are expressed as mean±s.e.m.

## Supplementary Material

10.1242/dmm.050148_sup1 Supplementary information
