# Supplementary material for: Variable body and tissue weight reporting in preclinical cachexia literature may alter study outcomes and interpretation
Source: Dis Model Mech. 2023 Jul 18;16(7):dmm050148. doi: 10.1242/dmm.050148 (PMC10387346; doi:10.1242/dmm.050148)
Supplement: Supplementary information [file dmm-16-050148-s1.pdf]

Dataset 1. C26 literature review body and tissue weight data extraction.

Click here to download Dataset 1

Dataset 2. C26 body and tissue weight data from animal study.

Click here to download Dataset 2

Disease Models & Mechanisms • Supplementary information
